# Supplementary material for: Development and validation of a prediction model for tocilizumab failure in hospitalized patients with SARS-CoV-2 infection
Source: PLoS One. 2021 Feb 23;16(2):e0247275. doi: 10.1371/journal.pone.0247275 (PMC7901750; doi:10.1371/journal.pone.0247275)
Supplement: S1 Table — (DOCX) [file pone.0247275.s001.docx]

S1 Table. Mean of biomarkers by case-control status in the unselected population

|  | **Case-Control status** | | | |
| --- | --- | --- | --- | --- |
| **Markers** | **Mech Ventilation-Death** | **Free of event** | **p-value^*^** | **Total** |
|  | N= 91 | N= 232 |  | N= 323 |
| ***Markers, Mean (SD)*** |  |  |  |  |
| Female, n(%) | 23 (25.3%) | 87 (37.5%) | 0.037 | 110 (34.1%) |
| Age, years | 65 (11) | 63 (13) | 0.212 | 63 (13) |
| SOFA Score | 3 (2) | 2 (1) | <.001 | 2 (2) |
| ***Po2/Fo2*** |  |  |  |  |
| Baseline | 220.9 (116.7) | 242.6 (101.6) | 0.117 | 236.6 (106.2) |
| Day 4 | 154.2 (101.6) | 246.0 (107.9) | <.001 | 224.6 (113.1) |
| Day 9 | 156.7 (96.4) | 249.0 (116.5) | <.001 | 224.3 (118.5) |
| Change Day 4 | -75.1 (126.0) | 17.8 (98.0) | <.001 | -4.2 (112.2) |
| Change Day 9 | -88.4 (125.8) | 23.2 (125.8) | <.001 | -6.9 (134.8) |
| ***Respiratory rate*** |  |  |  |  |
| Baseline | 24.8 (6.9) | 21.8 (5.7) | <.001 | 22.6 (6.2) |
| Day 4 | 24.3 (6.7) | 21.0 (7.7) | 0.020 | 21.6 (7.6) |
| Day 9 | 22.1 (5.4) | 19.9 (8.7) | 0.186 | 20.4 (8.1) |
| Change Day 4 | 0.9 (8.8) | -1.1 (9.3) | 0.252 | -0.7 (9.2) |
| Change Day 9 | -0.6 (8.5) | -1.9 (9.9) | 0.531 | -1.6 (9.5) |
| ***IL-6, pg/ml*** |  |  |  |  |
| Baseline | 426.7 (596.0) | 317.7 (427.6) | 0.433 | 335.0 (455.8) |
| Day 4 | 1451 (1094) | 797.8 (726.2) | 0.012 | 887.6 (810.4) |
| Day 9 | 979.3 (986.4) | 686.6 (755.2) | 0.471 | 710.5 (768.2) |
| Change Day 4 | 991.2 (981.9) | 460.3 (693.1) | 0.052 | 539.9 (757.9) |
| Change Day 9 | 271.1 (1432) | 217.5 (880.1) | 0.915 | 223.5 (928.4) |
| ***D-dimer, mg/dl*** |  |  |  |  |
| Baseline | 741.5 (1240) | 1281 (3292) | 0.343 | 1172 (2999) |
| Day 4 | 2624 (5614) | 2409 (4058) | 0.841 | 2440 (4294) |
| Day 9 | 2027 (2884) | 2419 (4099) | 0.697 | 2337 (3866) |
| Change Day 4 | 1348 (6368) | 556.8 (4697) | 0.548 | 679.1 (4965) |
| Change Day 9 | 1215 (3381) | 595.9 (6454) | 0.749 | 702.0 (6024) |
| ***hsCRP, mg/dl*** |  |  |  |  |
| Baseline | 12.8 (8.7) | 10.0 (7.7) | 0.007 | 10.8 (8.1) |
| Day 4 | 6.0 (6.1) | 3.3 (3.9) | <.001 | 4.0 (4.6) |
| Day 9 | 6.5 (10.2) | 1.4 (3.3) | <.001 | 2.5 (5.9) |
| Change Day 4 | -6.5 (9.4) | -6.8 (8.6) | 0.819 | -6.7 (8.8) |
| Change Day 9 | -6.3 (14.4) | -8.7 (8.7) | 0.197 | -8.2 (10.3) |
| ***Tot Lymphocytes, mg/dl*** |  |  |  |  |
| Baseline | 291.5 (672.8) | 604.3 (943.8) | 0.008 | 512.1 (883.1) |
| Day 4 | 540.2 (1127) | 800.4 (1137) | 0.130 | 737.2 (1138) |
| Day 9 | 784.2 (1504) | 1097 (1595) | 0.258 | 1021 (1575) |
| Change Day 4 | 23.0 (792.1) | 51.7 (726.7) | 0.806 | 44.7 (741.6) |
| Change Day 9 | -7.7 (466.0) | 381.8 (1005) | 0.021 | 288.0 (918.6) |
| ***Platelets, mg/dl*** |  |  |  |  |
| Baseline | 207.9 (92.7) | 244.9 (112.1) | 0.007 | 234.5 (108.1) |
| Day 4 | 253.8 (121.8) | 336.0 (143.8) | <.001 | 316.6 (143.0) |
| Day 9 | 266.3 (145.5) | 349.5 (141.8) | <.001 | 330.8 (146.5) |
| Change Day 4 | 46.1 (103.2) | 92.6 (98.0) | 0.002 | 81.4 (101.1) |
| Change Day 9 | 59.7 (145.1) | 122.2 (127.4) | 0.009 | 108.4 (133.6) |
| ^*^Chi2 for gender and unpaired t-test | | | | |
