# Supplementary material for: Development and validation of a prediction model for tocilizumab failure in hospitalized patients with SARS-CoV-2 infection
Source: PLoS One. 2021 Feb 23;16(2):e0247275. doi: 10.1371/journal.pone.0247275 (PMC7901750; doi:10.1371/journal.pone.0247275)
Supplement: S1 Fig — (DOCX) [file pone.0247275.s002.docx]

S1 Fig. AUC under the ROC analysis – training set

| *ROC Association Statistics* | | | | | | | |
| --- | --- | --- | --- | --- | --- | --- | --- |
| *ROC Model* | *Mann-Whitney* | | | | *Somers' D* | *Gamma* | *Tau-a* |
|  | *Area* | *Standard Error* | *95% Wald Confidence Limits* | |  |  |  |
| *Model* | 0.8974 | 0.0301 | 0.8384 | 0.9563 | 0.7947 | 0.7947 | 0.2200 |
| *ROC1* | 0.5000 | 0 | 0.5000 | 0.5000 | 0 | . | 0 |

| *ROC Contrast Test Results* | | | |
| --- | --- | --- | --- |
| *Contrast* | *DF* | *Chi-Square* | *Pr > ChiSq* |
| *Reference = Model* | 1 | 174.4378 | <.0001 |
