# Supplementary material for: Development and validation of a prediction model for tocilizumab failure in hospitalized patients with SARS-CoV-2 infection
Source: PLoS One. 2021 Feb 23;16(2):e0247275. doi: 10.1371/journal.pone.0247275 (PMC7901750; doi:10.1371/journal.pone.0247275)
Supplement: S2 Fig — (DOCX) [file pone.0247275.s003.docx]

S2 Fig. Average trend in PaO2/FiO2 ratio and biomarkers over day0-day9 by case-control status
