# Supplementary material for: Development and validation of a prediction model for tocilizumab failure in hospitalized patients with SARS-CoV-2 infection
Source: PLoS One. 2021 Feb 23;16(2):e0247275. doi: 10.1371/journal.pone.0247275 (PMC7901750; doi:10.1371/journal.pone.0247275)
Supplement: S3 Fig — (DOCX) [file pone.0247275.s004.docx]

**S3 Fig. AUC under the ROC for death in the training set and CV analysis**
