# Supplementary material for: Development and validation of a prediction model for tocilizumab failure in hospitalized patients with SARS-CoV-2 infection
Source: PLoS One. 2021 Feb 23;16(2):e0247275. doi: 10.1371/journal.pone.0247275 (PMC7901750; doi:10.1371/journal.pone.0247275)
Supplement: S4 Fig — (DOCX) [file pone.0247275.s005.docx]

S4 Fig. KM estimate of experiencing the composite event past day-4 window

| *Summary of the Number of Censored and Uncensored Values* | | | |
| --- | --- | --- | --- |
| *Total* | *Failed* | *Censored* | *Percent Censored* |
| 266 | 40 | 226 | 84.96 |
