# Supplementary material for: Development and validation of a prediction model for tocilizumab failure in hospitalized patients with SARS-CoV-2 infection
Source: PLoS One. 2021 Feb 23;16(2):e0247275. doi: 10.1371/journal.pone.0247275 (PMC7901750; doi:10.1371/journal.pone.0247275)
Supplement: Attachment — Submitted filename: Plos_Response_final.docx [file pone.0247275.s006.docx]

Modena 30/01/2021

To:

Academic Editor

PLOS ONE

Dear Editor,

We are very grateful for forwarding to us the reviewers’ comments to our paper PONE-D-20-35873 entitled: **Development and validation of a prediction model for tocilizumab failure in hospitalized patients with SARS-CoV-2 infection**

We here provide a point-by-point reply to the major and minor comments and we have incorporated the related changes in the manuscript.

Editor

1. Please ensure that your manuscript meets PLOS ONE's style requirements, including those for file naming.

**We checked the PLOS ONE style templates and we believe this revised version meets the journal requirements.**

2. We note that you have included the phrase “data not shown” in your manuscript. Unfortunately, this does not meet our data sharing requirements. PLOS does not permit references to inaccessible data. We require that authors provide all relevant data within the paper, Supporting Information files, or in an acceptable, public repository. Please add a citation to support this phrase or upload the data that corresponds with these findings to a stable repository (such as Figshare or Dryad) and provide and URLs, DOIs, or accession numbers that may be used to access these data. Or, if the data are not a core part of the research being presented in your study, we ask that you remove the phrase that refers to these data.

**We have now included a supplementary Table showing the results of the preliminary screening of the association between the markers value at baseline, day4 and day9 with the outcome in the whole dataset of 323 participants (Supplementary Table 1).**

3. PLOS requires an ORCID iD for the corresponding author in Editorial Manager on papers submitted after December 6th, 2016. Please ensure that you have an ORCID iD and that it is validated in Editorial Manager. To do this, go to ‘Update my Information’ (in the upper left-hand corner of the main menu), and click on the Fetch/Validate link next to the ORCID field. This will take you to the ORCID site and allow you to create a new iD or authenticate a pre-existing iD in Editorial Manager. Please see the following video for instructions on linking an ORCID iD to your Editorial Manager account: [https://www.youtube.com/watch?v=_xcclfuvtxQ](https://eur01.safelinks.protection.outlook.com/?url=https%3A%2F%2Fwww.youtube.com%2Fwatch%3Fv%3D_xcclfuvtxQ&data=04%7C01%7C%7C5c8bed073f7e4ebabb9b08d8c22db095%7C1faf88fea9984c5b93c9210a11d9a5c2%7C0%7C0%7C637472848775855057%7CUnknown%7CTWFpbGZsb3d8eyJWIjoiMC4wLjAwMDAiLCJQIjoiV2luMzIiLCJBTiI6Ik1haWwiLCJXVCI6Mn0%3D%7C1000&sdata=eBgXIfKKp%2BMeK6aTGwqaeDlhIZizcZy3B4eOfPmTigo%3D&reserved=0)

**Professor Cristina Mussini is the corresponding author who has a ORCID iD that is validated in Editorial Manager.**

 4. Please ensure that you refer to Figure 2 in your text as, if accepted, production will need this reference to link the reader to the figure.

**We apologize for the omission. Old Figure 2 is now Figure 1 and a reference has been added at line 205 of the revised version.**

Reviewers' comments:

**Comments to the Author**

Reviewer #1:

Dear Authors,

This study was interesting and brought further knowledge in COVID 19 especially in severe disease. My comments are mentioned below

1. Why do you choose female gender as the risk factor of severe. As we knew from many published trial men were strongly risk factors of severity in COVID 19

**We included sex in the score. The reviewer is absolutely right that males are at a higher risk than female. There was indeed a typo at lines 214 (in the revised version line 276) and 252 (in the revised version line 322) in the calculation of the risk score for a virtual patient and a score of +4 should be given to someone of male sex, not female (this is shown correctly in Table 4).**

2. Previous study discussed that Toci could improve biomarker of severe disease. (DOI: 10.1002/jmv.26698) This topic should be mentioned in the discussion. Although this study had novelty the first, you could discuss with the comparison of other virus disease or outbreak who develop risk scoring for treatment.

**We have now added the suggested reference and the following sentence has been incorporated in the Discussion section (lines 279-281):**

**“In support of the composition of our risk score, a recent meta-analysis showed that tocilizumab treatment was associated with reduction in a number of biomarkers including C-reactive protein (18).”**

**We indeed had built up our expertise in developing risk scores in the field of HIV resistance and we have also mentioned this in the Discussion (lines 337-339):**

**“To build the prediction model, we have used our previous experience in developing risk scores in the field of HIV resistance which was easily transferable to the field of COVID-19 research (23).”**

3. Although this scoring probability was internally statistically significant, this scoring system should be testing in external validation with higher and different population, and this should be mentioned in discussion or conclusion.

**We did validate the performance of the risk score on an external dataset of data collected at clinics not included in the training set although data were shown as supplemental material. We have now removed the performance of the model in training and replaced those results with a Figure showing the performance of the model in the external test set. We agree with the reviewer that the sample size of the test is small and we should aim to validate the score in the future on other larger sets. We have added a sentence in the Discussion regarding this future work (lines 381-382):**

**“However, the score should be further externally validated in the future using other test sets of data collected during the more recent waves of the pandemic.”**

4. Until now, we have still not known, what is the best time to give toci, may be this one of the factors, why the results of the study were conflicting, and previous study was using Chloroquine which data from Recovery showed harmful. This population also still used Chloroquine, this issue should be considered.

**Indeed, tocilizumab was added to chloroquine in most participants as it was standard of care at the time but it was an early use and the drug was discontinued promptly as it did not show much benefit. Unfortunately, as few patients were not using chloroquine, the effect of tocilizumab affected by use of this drug could not be tested in our study. We have now specifically mentioned chloroquine in line 385 of the revised version. We agree that trials addressing the question ‘when is best to treat with tocilizumab’ are lacking. However, recent evidence from trials have led to a change of treatment guidelines in the UK, that are now recommending the use of tocilizumab in critical patients. We have planned a trial at University of Modena and Reggio Emilia to specifically address this question.**

 Reviewer #2:

The Authors here presented a well conducted and clear study. The statistical method is clear exposed and nicelt designed. All the data are clear. The study limitations are well described in the discussion. Only few comments:

- Line 41: there is another meta-analysis you should cite to reinforce this point: Mortality in tocilizumab-treated patients with COVID-19: a systematic review and meta-analysis. Clin Exp Rheumatol. 2020 Nov-Dec;38(6):1247-1254. Epub 2020 Dec 3. PMID: 33275094.

**The literature on tocilizumab indeed grows very quickly. We thank you the reviewer for pointing out the new meta-analysis. We have revised the state of art on tocilizumab treatment and added some more recent published meta-analyses and randomized trials. The following sentences have been added or modified in the Introduction:**

**“These results were confirmed by 2 more recently published meta-analyses including a larger number of studies (8,9).” – lines 87-88**

**“A pharmaceutical company press releases showed no evidence for clinical improvement and reduced risk of mortality when comparing patients treated with tocilizumab vs. placebo in the double-blind COVACTA trial, but final results remain unpublished (10). Similar negative results were obtained prescribing tocilizumab to patients with mild to moderate COVID19 pneumonia in the attempt to prevent the cytokine storm (11, 12).” – lines 90-95**

**“More recently, the REMAP_CAP trial conducted in critically ill patients showed a reduction in mortality in patients receiving either tocilizumab or sarilumab. As a consequence of these results, tocilizumab is now recommended for use in the clinics for patients in critical conditions in the UK (15).” – lines 102-114**

- Line 44: to date this affermation is not true, there is at least one RCT you should cite: Tocilizumab Trial Investigators. Efficacy of Tocilizumab in Patients Hospitalized with Covid-19. N Engl J Med. 2020 Dec 10;383(24):2333-2344. doi: 10.1056/NEJMoa2028836. Epub 2020 Oct 21. PMID: 33085857; PMCID: PMC7646626.

**See our response to previous point. At the time of submission (late November 2020), none of the ongoing trials on tocilizumab had been published. We thank you the reviewer for point out the trial published on NEJM. We have revised sentences concerning the state of art on tocilizumab treatment and added the reference for this and other recent published trials.**

- Correct some typo errors

**We have carefully checked the whole text for typos and corrected them.**

Reviewer #3:

To properly evaluate the performance of a prediction model, only using an independent testing dataset is meaningful. It’s great there is an independent dataset available in the study. The matter is then to describe the performance of the model properly and objectively—build the model using the training dataset and evaluate its performance using the testing dataset. Please remove the results of cross-validation on the same dataset to avoid the unnecessarily exaggeration of the model performance.

**We agree with the reviewer that the performance on the test set is key. We have therefore removed Figure 1 of the performance on training (which we agree is misleading) and replaced with Supplementary Figure 3 with the performance on test. Regarding the data obtained in cross-validation, we respectfully disagree with the reviewer. Cross-validation is a good estimator of the extra-sample error distribution and especially in our case, in which the sample size of the test set is small, it is a crucial step and represents even a more robust validation if no population shift or selection bias is expected. Indeed, the TRIPOD statement for model predictions indicate Internal validation as a necessary part of model development [Steyerberg EW: Clinical Prediction Models: A Practical Approach to Development, Validation, and Updating. New York: Springer; 2009.; Collins GS, Reitsma JB, Altman DG, Moons KG. Transparent reporting of a multivariable prediction model for individual prognosis or diagnosis (TRIPOD): the TRIPOD statement. BMJ. 2015 Jan 7;350:g7594.].**

**We hope that this revised version of the paper is now suitable for publication in PLOS ONE and we look forward to hearing back from you.**

**Best regards,**

**Prof. Cristina Mussini**
